# Population extinctions driven by climate change, population size, and time since observation may make rare species databases inaccurate

**DOI:** 10.1371/journal.pone.0210378

**Published:** 2019-10-17

**Authors:** Thomas N. Kaye, Matt A. Bahm, Andrea S. Thorpe, Erin C. Gray, Ian Pfingsten, Chelsea Waddell

**Affiliations:** 1 Institute for Applied Ecology, Corvallis, Oregon, United States of America; 2 Department of Botany and Plant Pathology, Oregon State University, Corvallis, Oregon, United States of America; 3 Nonindigenous Aquatic Species Program, Cherokee Nation Technology Solutions, Wetland and Aquatic Research Center, Gainesville, FL, United States of America; 4 Wildlife/Botany & Fisheries/Aquatics Data Coordinator, Branch of Biological Resources, United States Bureau of Land Management, Oregon State Office, Portland, OR, United States of America; University of Bucharest, ROMANIA

## Abstract

Loss of biological diversity through population extinctions is a global phenomenon that threatens many ecosystems. Managers often rely on databases of rare species locations to plan land use actions and conserve at-risk taxa, so it is crucial that the information they contain is accurate and dependable. However, small population sizes, long gaps between surveys, and climate change may be leading to undetected extinctions of many populations. We used repeated survey records for a rare but widespread orchid, *Cypripedium fasciculatum* (clustered lady’s slipper), to model population extinction risk based on elevation, population size, and time between observations. Population size and elevation were negatively associated with extinction, while extinction probability increased with time between observations. We interpret population losses at low elevations as a potential signal of climate change impacts. We used this model to estimate the probability of persistence of populations across California and Oregon, and found that 39%-52% of the 2415 populations reported in databases from this region are likely extinct. Managers should be aware that the number of populations of rare species in their databases is potentially an overestimate, and consider resurveying these populations to document their presence and condition, with priority given to older reports of small populations, especially those at low elevations or in other areas with high vulnerability to climate or land cover change.

## Introduction

Population extinctions are a major threat to plants, leading to range contractions, fragmentation and isolation (e.g., [1–4]), which together reduce the abundance of species. As Darwin [5] pointed out, rarity is a precursor of extinction. Orchids in particular face a global conservation risk with high species diversity but also a high rate of species that are rare or threatened with extinction [6–12], and rare orchids are likely to need aggressive conservation actions to prevent their extinction [13]. Nearly half of the genus *Cypripedium* may be threatened and in need of protection if the species are to survive in the wild [14]. Therefore, accurate assessments of the number of populations of a rare species and its major threats are crucial to conservation planning and resource allocation for recovery actions [15, 16].

Several processes can contribute to rare plant population extinctions, including habitat loss, interactions with invasive species, changes in disturbance frequency, etc. [17]. Climate change in particular is affecting species ranges globally [18], with organisms shifting toward higher latitudes [19] and elevations [20]. For example, plant ranges in western Europe have moved upslope at 29 m/decade over the last century [21] and in California at similar rates [22]. Climate change effects on temperature and moisture may threaten plant diversity in Europe, especially in mountains [23]. Low-elevation populations of organisms can be especially at risk of extirpation as climatic conditions change and force upslope range shifts [24]. Any contraction in the range of a rare species can have significant effects on its long term conservation and viability.

The number of individuals present can also affect the viability of plant populations, with small populations having greater risk of extirpation. In general, the extinction probability of a population increases as population size decreases [25, 26]. Small populations may be at greater risk of extinction because of several factors, including losses in reproductive individuals [27], Allee effects [28], declines in seed production [29] and viability [30], loss of genetic diversity [31] and accumulation of genetic load [32], and demographic stochasticity [33]. In empirical studies that surveyed the same locations of multiple plant species over several years in Germany [4] and the Swiss Jura Mountains [34], extinction rates were found to be higher for small populations. And although population size may be a strong predictor of population vulnerability, passage of time can compound the likelihood of extinction because as more time passes in stochastic environments the chances that a population will fall to zero increase [25, 26].

Taken together, climate change, population size, and time since observation create considerable uncertainty regarding the current status of wild plant populations recorded in various rare species databases. Several US agencies and organizations (e.g., US Bureau of Land Management, US Fish and Wildlife Service, US Forest Service, NatureServe, state Natural Heritage Programs) maintain databases of rare plant occurrences and many of these occurrences may not have been visited recently. Therefore, the number of populations in the wild of some species could be smaller than the number listed in databases due to extinctions that have not yet been detected. Increasing our ability to estimate the number of populations that remain extant or have gone extinct in these data bases will improve conservation planning for rare species. We used information on repeated surveys in California and Oregon for a rare but widespread orchid, *Cypripedium fasciculatum* (clustered lady’s slipper), to test the hypothesis that extinction probability is affected by elevation, population size, and time since observation. We applied the resulting model to populations in land management databases in Oregon and California, specifically the Geographic Biotic Observations (GeoBOB) data base maintained by the US Bureau of Land Management and the US Forest Service Natural Resource Information System (NRIS-Terra), to estimate the number of populations that are still extant.

## Materials and methods

### Study species

*Cypripedium fasciculatum* (clustered ladies slipper) occurs in scattered population centers in western North America in California, Oregon, Washington, Idaho, Montana, Utah, Wyoming and Colorado. In California and Oregon, this taxon occurs predominantly in the Klamath-Siskiyou Mountains and Sierra Nevada Mountains. The United States Forest Service (USFS) considers it to be a Sensitive Species and the Bureau of Land Management (BLM) lists it as a Bureau Sensitive Species (designations that indicate population viability is of concern and the species may need special management consideration), and it is considered globally secure because of its widespread geographic range and abundance in some states [35]. In California and Oregon the species is most often found on north facing slopes in mixed coniferous forests of >60% canopy closure [36]. *Pseudotsuga menziesii* is the most common associated tree, but other frequently noted forest components include *Abies concolor*, *Cornus nuttallii*, *Pinus lambertiana*, and *Calocedrus decurrens*. Clustered lady's slipper is known to occur in California and Oregon at elevations from about 180 to nearly 2000 m. The species has a complex life-history and depends on specific mycorrhizal fungi [37], which may affect its seed germination and growth. Mycorrhizal fungi may determine where and in which specific habitats this orchid can grow and how it responds to disturbance, but little information is available on the fungi, their requirements, associated tree species, and their function in forest ecosystems [36].

### Data sources

We compiled repeated-survey data from multiple sources to test for effects of elevation, time between surveys, and population size on extinction probability. The sources of these resurvey data were from an assessment of the conservation status of *C*. *fasciculatum* in California that reviewed available records (78 sites) for the species throughout that state [36] and from repeated surveys in southwestern Oregon (127 sites) [38] conducted on federal lands. Both resurvey data sources (205 populations combined) included sites revisited at least once and documented site location, elevation, population size, and years between surveys. We used information on population size from the first survey, and time between first and last surveys was calculated as the number of years between the first and last (most recent) survey. The last survey was used to score each population as either extant or extinct (no individual plants found at the site). The time between surveys ranged from 1 to 29 years. While most observers censused populations, some (particularly in the California portion of the data set) estimated population size, and when this occurred we used the highest integer reported for a population during the first survey. For example, if 50–100 plants were reported, we used 100 to be conservative. If the number was vague (e.g., 75+, >30, or ca. 50) we used the actual integer listed (75, 30, or 50, respectively). Populations used in the analysis varied in size from 1 to 1084 individuals. *C*. *fasciculatum* plants that were single stems or clumps were considered individuals (following [39]).

### Population viability analysis

We used logistic regression ([40], glm in R stats package) to construct models to estimate extinction probability. The response variable was population status at the most recent visit (a binomial response, either extinct or extant) and predictor variables were size of the population at the first survey, number of years between the first and last survey, and elevation (m) of the population. Population size was log-transformed in the logistic regression model to meet assumptions of normality. We used a model selection routine comparing Bayes Information Criterion [41] values among models with all combinations of predictor main effects and interactions, and we choose the most parsimonious model based on the smallest BIC value. All analyses were performed in R 3.6.0 [42]).

### Estimating number of extant populations

To estimate the number of populations of *C*. *fasciculatum* recorded as still extant in the land management databases, GeoBOB and NRIS-Terra, for California and Oregon, we applied our selected final model for predicting extinction probability to the 2896 populations recorded in those databases based on their size, years since the last survey, and elevation. Predicting average population survival is theoretically possible using regression coefficients, however prediction uncertainty in logistic regression is not derived from coefficient uncertainty like in linear regression, and instead can be empirically derived. Logistic regression prediction uncertainty, which assumes a binomial distribution, is inaccurately wide, even if predictions are converted to log-odds ratios, when theoretically estimated using coefficient uncertainty, which assumes a Gaussian distribution (see our R script for example). To empirically estimate uncertainty around predicted survival of all populations, we bootstrapped the coefficients in our chosen model by randomly selecting 205 populations from our model building data set, with replacement, and estimating the logistic regression coefficients at each iteration [43]. For each bootstrapped set of coefficients, we calculated the extinction probability of each population in the land management databases, subtracted from one and summed those probabilities to estimate the number of extant populations, and repeated this bootstrap process 10,000 times to estimate 95% confidence limits (i.e., prediction uncertainty). We performed this analysis in R 3.6.0 [42].

## Results

### Population viability analysis

**Fig 1:**
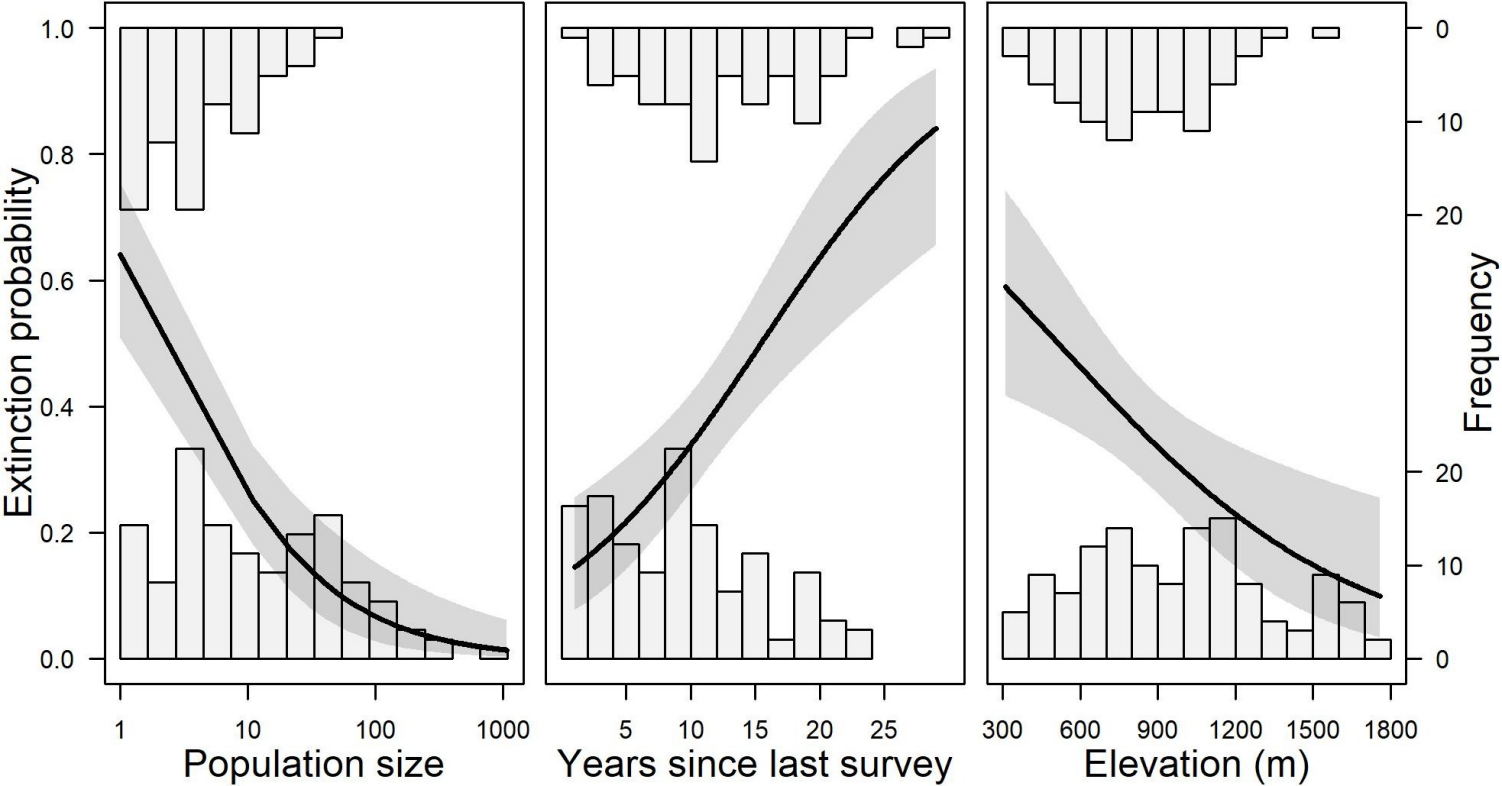
Estimated extinction probability of *Cypripedium fasciculatum* as a function of population size (left), years between surveys (center), and elevation (right). Shadings around each line represent 95% confidence intervals. Histograms indicate the frequency (labeled on right axis) of mortality (top axis) and survival (bottom axis).

Of the 205 populations in our data sets, 34% were no longer present when resurveyed. The most parsimonious extinction model based on largest ΔBIC included only the main effects of population size, time between surveys, and elevation. Each of these factors was significant in the final model for predicting extinction probability of populations (Table 1). The general linear model suggested that small populations had a greater probability of extinction than large populations, and extinction probability was near zero for populations with >100 individuals (Fig 1, left). Extinction probability increased as the time between surveys increased (Fig 1, center). Finally, populations at lower elevations were more likely to go extinct than those at high elevations (Fig 1, right).

**Table 1:** Generalized linear logistic regression model for factors affecting the probability of population survival for *C*. *fasciculatum* with coefficient estimates, standard errors, z scores, and *p*-values. The resulting model takes the form log_*e*_(y/(1—y)) = β_0_ + β_1_X_1_ + β_2_X_2_ + β_3_X_3_.

Factor	Estimate	Standard Error	z score	*p*-value
β_0_ - (Intercept)	0.93	0.58	1.60	0.110
β_1_ - Starting population size	-0.70	0.14	-4.93	<0.001
β_2_ - Years between surveys	0.12	0.029	4.28	<0.001
β_3_ - Elevation	-0.0018	0.00059	-2.98	0.003

### Estimating extant populations

**Fig 2:**
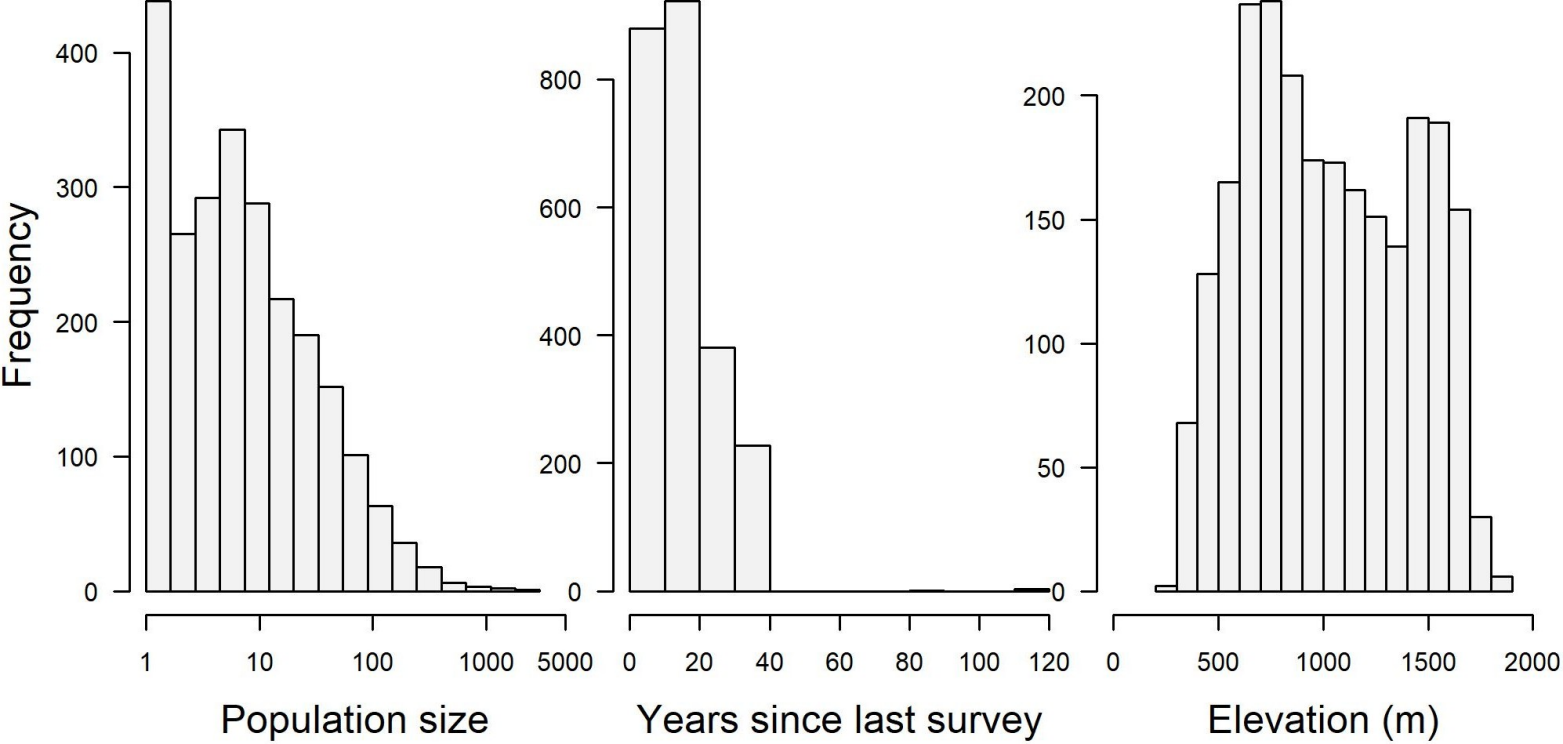
Frequency distribution of population size, years since last survey, and elevation for populations in the land management databases.

**Fig 3:**
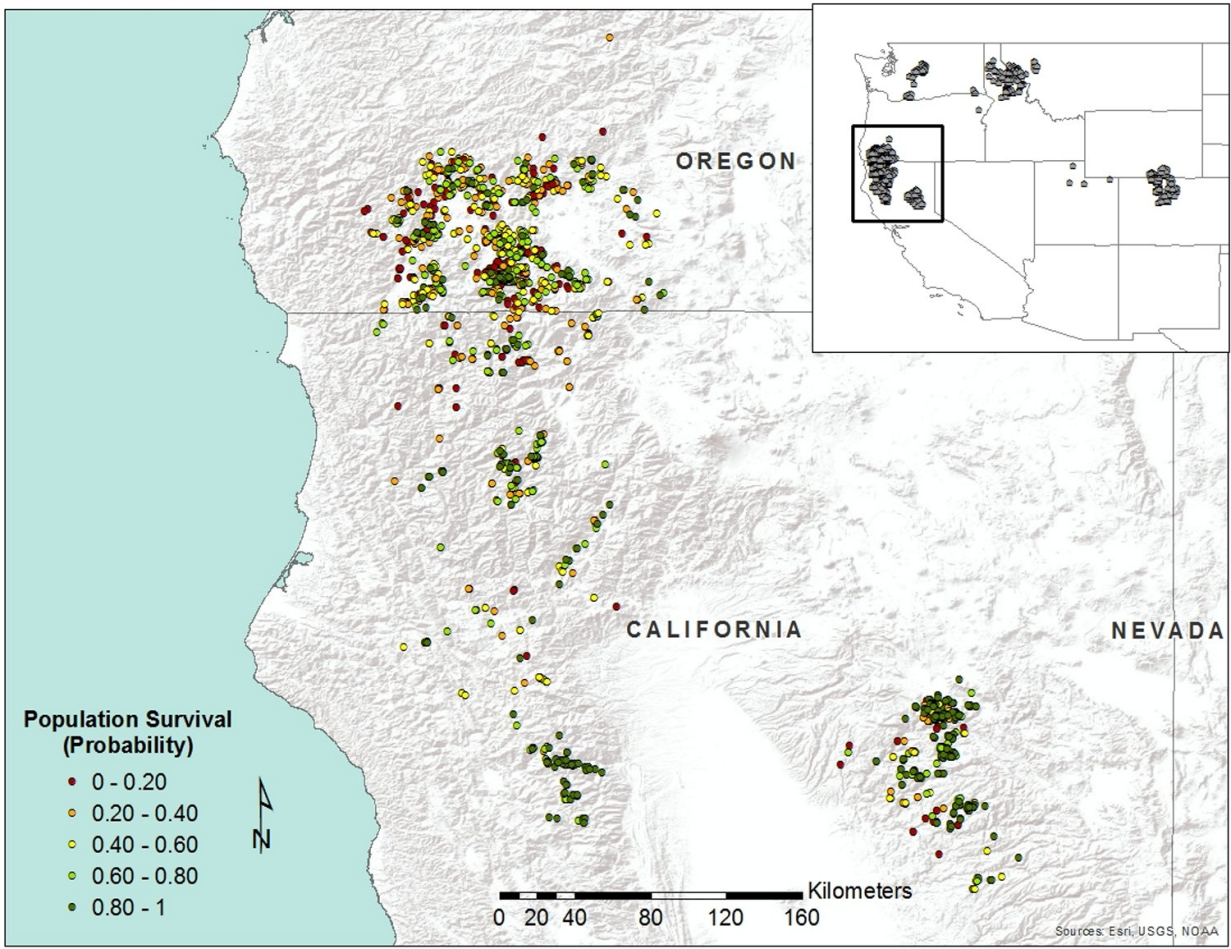
Distribution of *Cypripedium fasciculatum* in California and Oregon showing the probability of persistence estimated from population size, time since observation, and elevation. Insets show known locations of the species in the western United States (data from GeoBOB and NRIS-Terra) and a photo of the plant from southwestern Oregon.

A total of 2415 populations with one or more plants were reported in the land management databases for Oregon and California. An additional 426 populations were reported as already extinct by 2016. Populations in those land management databases ranged in size from 1 to 1859 individuals, years between surveys from 1 to 115, and elevations from 234 to 1851 (Fig 2). The mean population size was 25 (95% CI ± 1.6). We estimated that of the 2415 populations reported as extant, only 1,317 (95% bootstrapped quantiles: 1,164–1,476) were likely still present. This is equivalent to an overall extinction rate of 45% (95% bootstrapped quantiles: 39%-52%). The predicted probability of population survival varied widely across the landscape in California and Oregon, with some population centers showing greater potential for population extinction than others (Fig 3). For example, populations in southwestern Oregon had a predicted extinction rate of 57% (49%– 64%) of 1258 reports compared to 33% (25% - 41%) of 1157 records in California. This difference was driven in our model by the generally lower population sizes in Oregon (mean: 12.7 95% CI: ± 1.5) than California (37.9 ± 6.6) and lower elevations of populations in Oregon (757.2m ± 13.4m) than California (1319.2m ± 15.4m). Years between observations did not differ between states, averaging 15.4 years overall (± 0.39).

## Discussion

We found that population size, time between surveys, and elevation predicted extinction in *Cypripedium fasciculatum*. When these factors were used to model the persistence of wild populations, we found that only 55% of populations reported in land management databases (GeoBOB and NRIS-Terra) for California and Oregon are likely still present on the landscape. Extinction rates are predicted to be higher in Oregon than in California, primarily due to the lower average population size and elevation there. Many orchid species have populations with a wide range of sizes [44], and small average population sizes are common. The average population size of *Cypripedium fasciculatum* in California and Oregon was 25 individuals. The average population of *C*. *kentuckiense* has 40 individuals, *C*. *calceolus* in Europe generally has populations with fewer than 100 plants, and *C*. *dickensonianum* occurs as small colonies or single individuals [45]. As population size declines in orchid species, gene flow by pollen may decline [46], inbreeding may increase [47], pollination, fruit set and seedling recruitment may decrease [48], genetic drift may increase [49], and genetic diversity may decline [50]. Transition matrix models of *C*. *calceolus* [51] indicate extinction probability over a 100 year period in populations with 10 plants is 37%, and in populations with 5 plants it increases to 67% without disturbance. In populations where flowers are removed or plants are dug up, extinction probability rapidly approaches 100%. The typically low population size in *C*. *fasciculatum* was a major contributor to the high rate of predicted extinctions we have shown empirically for the species.

Population extinction probability was associated with time between surveys in *C*. *fasciculatum*. In stochastic environments, even populations with stable intrinsic population growth rates are vulnerable to extinction, and this vulnerability increases with time [25, 26]. In populations with declining growth rates, the rate of extinction will be even faster. Therefore, as time between surveys increases, population extinction should also increase, especially for small populations. Surprisingly, time between surveys had no significant effect on probability of extinction in eight rare plants in Germany [4], but the study was conducted over a relatively short period (ten years).

We speculate that negative impacts from climate change might already be apparent for *C*. *fasciculatum* through extinction of low elevation populations. Loss of low elevation populations may be expected when climates warm to the point that populations can no longer survive in the hotter portions of their range. For example, loss of butterfly species at low elevations has been attributed to warming trends in Spain [52]. Our findings with *C*. *fasciculatum* are generally consistent with orchid responses to climate change in North America and elsewhere. Documented declines of species in the Orchidaceae in eastern North America appear to be related, at least in part, to an inability of these species to alter their phenology, particularly flowering time, as climate has warmed over the last century and a half [53]. Climate change appears to be a threat to orchids in Mexico [54], and orchids in general appear to be highly vulnerable to climate change in China [55]. In contrast, orchids were more likely to increase abundance in mediterranean France from 1886–2001 compared to many other plant taxonomic groups [56]. Precipitation appears to be a strong driver of plant survival in *C*. *reginae* [57], making the species vulnerable to changes in regional climate. And it is clear that climate has changed recently and is forecasted to change further in California and Oregon, in part due to warming and drying that, when combined, exacerbate moisture deficits and increased evaporative demand (e.g., [58]). Changes in land use and land cover could also explain the loss of low elevation populations [59], but the lands on which this study derived its supporting data are public property that is remote, often difficult to access, and largely sheltered from development and urbanization. Also, the status of this plant as a sensitive species has led to the protection of most populations on public land from timber harvest, road building, and other land actions for the period of this study. Nitrogen deposition has also contributed to large scale landscape changes that affect plant distributions [60], but the populations examined here are not directly downwind from urban centers with high N production and receive most of their precipitation from low-N air masses blown in from the Pacific Ocean.

Resurveys of plant populations and communities can provide substantial insights into the nature and causes of changes that occur in the natural world over time [61–64]. Even so, there are some limitations to our estimates of extinction probability of *C*. *fasciculatum* in this study. Repeated surveys may fail to relocate previously documented populations even when they are still present [65–67] if the survey is not sufficiently thorough. The datasets we used contained information on population resurveys that were carefully conducted by trained botanists with precise location information, but the possibility remains that some extant populations may have been missed. This could be aggravated by individual plant dormancy, which would make plants very difficult to detect during surveys, and if all plants in a population were dormant at the same time–a possibility that increases as population size declines–whole extant but dormant populations could be falsely classified as extinct. Dormancy (years when no living tissue is visible above ground) is not uncommon in terrestrial orchids [68], including *Cypripedium* [39, 69–73]. *Cypripedium reginae*, for example, may be dormant for up to four years [57]. On the other hand, dormancy is associated with decreased orchid reproduction [74] and survival [75], and if all individuals in a population were dormant, the population might already be close to extinction. These factors suggest that although we could have overestimated extinction probability [65] due to dormancy, this same dormancy could suggest increased plant vulnerability. Either way, we are unable to quantify this potential bias in our results given the available data.

Because orchids depend on fungi, at least in the early stages of plant development, the presence of appropriate fungi and the environmental factors that affect them may in turn determine the growth and survival of many orchids [76], including *C*. *fasciculatum* populations. Soil and topography, and especially temperature and moisture are the most important factors that control orchid distribution and survival [77], and this may be due to the influence of these factors on mycorrhizal fungi. *Cypripedium* spp. are associated with fungi in the Sebacinaceae, Ceratobasidiaceae, and especially the Tulasnellaceae [37]. The degree of specificity of orchids with fungi is significant because orchids with highly specific associations may be more sensitive to disturbance and environmental change than generalist species [78]. Further, climate and fungal symbionts of orchids may interact to shape the evolutionary response of specific vital rates to climate change, such as sprouting after dormancy [79].

Seed dispersal can lead to the recruitment of new populations on the landscape. Some or all of the extinctions we observed and modeled could be offset but the establishment of new wild populations. However, data from a portion of the geographic area of our study system suggests that the rate of establishment of new populations is approximately 0.000018 populations/ha/yr, while extinction is 2.8 times that rate (0.00005 populations/ha/yr) over the same time period (S3 File). Therefore, although new populations may continue to appear on the landscape, they may not balance the losses from extinctions. Also they are not readily recorded unless they are discovered during new searches, contributing to the lack of reliability of land management databases.

### Implications for conservation

This study demonstrates the need for additional and more frequent surveys of rare plant populations to improve the reliability of information in databases used by land management agencies. Land managers who make decisions on how best to conserve rare species often base their decisions in part on the abundance and distribution of those organisms as reported in databases. However, many reported populations may no longer be extant. Managers should be aware that the number of populations of rare species in their databases is potentially an overestimate, and consider resurveying populations in databases to document their presence and condition, with priority given to older reports of small populations, especially those at low elevations or other areas with high vulnerability to change in climate or land use. Species like *C*. *fasciculatum* may be candidates for assisted migration [80–82] as their low-elevation populations experience extinction and if expansion or colonization at higher elevation locations does not occur naturally. We suggest that development of propagation and planting techniques (e.g., [83–85]) to allow for intervention is warranted, and needs to consider the fungal dependency of this rare orchid [86].

## Supporting information

S1 File Data from resurveys of 205 populations of *Cypripedium fasciculatum* in Oregon and California with elevation (m) of observation, years since original observation, presence or absence at last observation, original population size (oldsize), and population size at last observation. (CSV)

S2 File Data from land management databases of 2415 populations of *Cypripedium fasciculatum* in Oregon and Washington with population size, elevation (m), year of observation, and years since observation. (CSV)

S3 File Methods and results of new population recruitment analysis for *Cypripedium fasciculatum* in southwestern Oregon, USA. (PDF)
