# Supplementary material for: Population extinctions driven by climate change, population size, and time since observation may make rare species databases inaccurate
Source: PLoS One. 2019 Oct 17;14(10):e0210378. doi: 10.1371/journal.pone.0210378 (PMC6797133; doi:10.1371/journal.pone.0210378)
Supplement: S3 File — (PDF) [file pone.0210378.s003.pdf]

New Population Recruitment

Methods

C. fasciculatum surveys and sites from the GeoBOB database were used to determine whether new populations were discovered in areas where the species was previously absent. To determine the number of new *C. fasciculatum* populations that were discovered within areas that had previously been surveyed but the species was not found, we used *ESRI ArcGIS* software to isolate vascular plant surveys within the established survey window (April 1 – August 1) that had been entered in the GeoBOB database within BLM Medford District as of July 2017 (n=10,126). Negative Surveys (n=9,897) for *C. fasciculatum* were then spatially intersected with known sites (n=1,323). The resulting 239 records were then evaluated to determine whether each site was discovered after the negative survey had been conducted and if the additional documentation about that survey supported that it was negative. Each site was then cross checked with the Oregon Biodiversity Information Center (ORBIC) database. The number of surveys that did not result in a positive finding prior to the *C. fasciculatum* population being discovered, the number of years between those negative surveys and the site being found, population size, and elevation were then summarized. Additionally, the number of surveys conducted between 2006 and 2016 and the area surveyed for CYFA were calculated. Finally, the rate of detection for new populations of *C. fasciculatum* was calculated. We also calculated the rate of extinction on the same scale by using estimates of population loss in the Medford District from the period of 2006 to 2015.

Results

We found that 17 *C. fasciculatum* populations were detected after being surveyed between 2006 and 2017, or 1.8% of BLM's Medford District's 967 current sites were found after being surveyed two or more times. Of those 17 *C. fasciculatum* populations, there was an average of 4.3 (±2.9) years between the initial survey and the new population being found. Of those 17 sites, 15 had been previously surveyed once and 2 had been previously surveyed twice. These newly discovered populations ranged in size of from 1 individual to 36 individuals and were found at approximately 2358 (1040-3450) ft elevation. Between 2006 and 2016, a total of 5,389 surveys and 95,103 hectares were surveyed for *C. fasciculatum* within the April 1 to August 1 survey window. This equates to a population recruitment rate of 17 populations on 95,103 ha in ten years, or 0.000018 populations/ha/y. Population extinction across the 351,429 ha of the Medford District was estimated at 158 in 9 y, for an extinction rate of 0.00005 populations/ha/y.
